# Preparation and Material Application of Amylose-Polymer Inclusion Complexes by Enzymatic Polymerization Approach

**DOI:** 10.3390/polym9120729

**Published:** 2017-12-18

**Authors:** Saya Orio, Kazuya Yamamoto, Jun-ichi Kadokawa

**Affiliations:** Department of Chemistry, Biotechnology, and Chemical Engineering, Graduate School of Science and Engineering, Kagoshima University, 1-21-40 Korimoto, Kagoshima 860-0065, Japan; k3951278@kadai.jp (S.O.); yamamoto@eng.kagoshima-u.ac.jp (K.Y.)

**Keywords:** amylose host, enzymatic polymerization, hierarchical structured material, inclusion complex, vine-twining polymerization

## Abstract

This review presents our researches on the preparation and material application of inclusion complexes that comprises an amylose host and polymeric guests through phosphorylase-catalyzed enzymatic polymerization. Amylose is a well-known polysaccharide and forms inclusion complexes with various hydrophobic small molecules. Pure amylose is produced by enzymatic polymerization by using α-d-glucose 1-phosphate as a monomer and maltooligosaccharide as a primer catalyzed by phosphorylase. We determined that a propagating chain of amylose during enzymatic polymerization wraps around hydrophobic polymers present in the reaction system to form inclusion complexes. We termed this polymerization “vine-twining polymerization” because it is similar to the way vines of a plant grow around a rod. Hierarchical structured amylosic materials, such as hydrogels and films, were fabricated by inclusion complexation through vine-twining polymerization by using copolymers covalently grafted with hydrophobic guest polymers. The enzymatically produced amyloses induced complexation with the guest polymers in the intermolecular graft copolymers, which acted as cross-linking points to form supramolecular hydrogels. By including a film-formable main-chain in the graft copolymer, a supramolecular film was obtained through hydrogelation. Supramolecular polymeric materials were successfully fabricated through vine-twining polymerization by using primer-guest conjugates. The products of vine-twining polymerization form polymeric continuums of inclusion complexes, where the enzymatically produced amylose chains elongate from the conjugates included in the guest segments of the other conjugates.

## 1. Introduction

**Figure 1:**
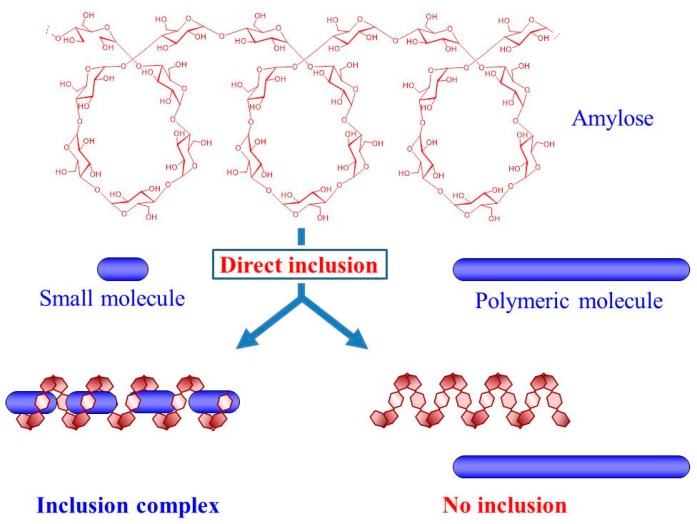
Amylose forms inclusion complex with relatively low molecular weight (small) hydrophobic molecule but largely, does not form it with polymeric molecule.

Biopolymers such as polysaccharides, proteins, and nucleic acids are common in Nature and play important in vivo roles [1,2,3]. The biological functions of polymers such as polysaccharides are achieved through both their primary chemical structures and controlled higher-order structure. Amylose is a natural linear polysaccharide with a left-handed helical conformation, which consists of glucose residues linked through α(1→4)-glycosidic linkages [3]. It is a main component of starch and functions as an energy storage molecule with the other component of starch, amylopectin. The seclusion of hydroxy groups in the glucose units to the outer side of the helix creates a hydrophobic cavity inside the helices. Therefore, amylose can act as a host to form host-guest inclusion complexes with hydrophobic guest molecules of low molecular weight through hydrophobic interactions (Figure 1) [4,5]. In addition to the traditional functions of these inclusion complexes, they can be manipulated to form higher-order materials with extended functionalities and properties suitable for specific practical applications. Polymeric guest molecules with high molecular weights are promising candidates for complexation with amylose, compared to low molecular weight guests, to achieve new functionalities. However, a limited number of studies have been reported on the complexation of amylose and polymeric guest molecules (Figure 1). Because weak hydrophobic interactions drive the incorporation of guest molecules into the amylose cavity, amylose does not have the ability to encapsulate large polymeric guests into its cavity. For the direct incorporation of polymeric guests, hydrophilic groups can be introduced at the polymer chain ends, which enhance the degree of complexation in aqueous media [6,7]. Additional methods to directly form amylose-polymer inclusion complexes include inclusion polymerization and guest-exchange approaches [8,9,10].

**Figure 2:**
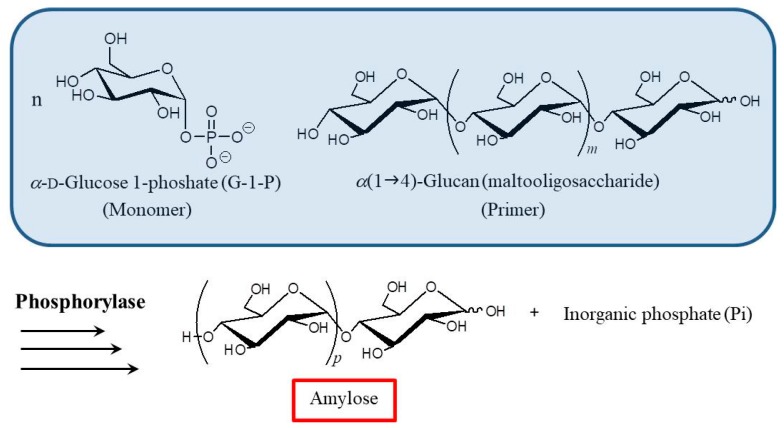
Phosphorylase-catalyzed enzymatic polymerization to produce amylose with well-defined structure.

**Figure 3:**
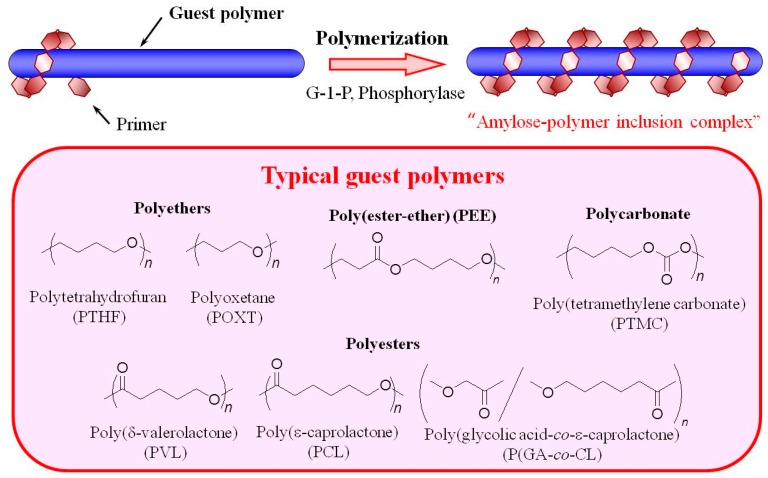
Image for vine-twining polymerization and typical guest polymers.

Recently, it has been accepted that the enzymatic approach is a powerful tool to precisely synthesize polysaccharides [11,12,13,14,15,16], and amylose with a well-defined structure can be synthesized by phosphorylase-catalyzed enzymatic polymerization of α-d-glucose 1-phosphate (G-1-P) and maltooligosaccharide as a monomer and primer, respectively (Figure 2) [17,18,19,20]. The polymerization is analogous to living polymerization because there are no significant termination or chain-transfer reactions. Accordingly, the molecular weight of the produced amylose can be controlled by monomer/primer feed ratios and typically result in narrow distributions [21]. By means of this enzymatic polymerization for the direct production of amylose, we developed an efficient method for the formation of inclusion complexes with synthetic polymers. The elongation of the short α(1→4)-glucan (maltooligosaccharide) to the longer α(1→4)-glucan (amylose) is considered to provide sufficient dynamic field for more facile complexation of polymeric guests compared to the direct complexation between the polymeric host (amylose) and guest [22,23,24,25,26,27]. The polymerization propagation is similar to the way that the vines of plants grow, twining around a rod (Figure 3). Accordingly, we proposed that this polymerization method for the production of amylose-polymer inclusion complexes should be called “vine-twining polymerization”. Furthermore, the vine-twining approach has been applied to the dynamic preparation of supramolecular networks/higher-order materials [28]. This review summarizes the preparation and material application of amylose-polymer inclusion complexes fabricated by the vine-twining polymerization approach achieved in our research group.

## 2. Preparation of Amylose-Polymer Inclusion Complexes by Enzymatic Polymerization Filed (Vine-Twining Polymerization)

In the following section, we discuss typical characteristics required by guest polymers to dynamically form inclusion complexes with amylose in vine-twining polymerization. As mentioned previously, hydrophobicity is required for inclusion complexation in the cavity of amylose. As vine-twining polymerization is conducted in an aqueous buffer solvent, the guest polymers must be able to be dispersed in aqueous media. Therefore, relatively polar groups, such as ethers and esters, should be present in the main-chain of the guest polymers. The guest polymer must also be slender without bulky substituents because of the cavity size of the amylose helix is not sufficiently large to encapsulate most bulky molecules. Based on the above features, hydrophobic synthetic polymers shown in Figure 3 have been found to act as guest polymers for the formation of inclusion complexes with amylose in vine-twining polymerization.

The first example of vine-twining polymerization was reported using polytetrahydrofuran (PTHF) as a hydrophobic guest polyether [29,30]. The structure of PTHF has been identified as suitable for guest polymers because it is generally hydrophobic, but includes relatively polar ether groups without any side groups. When the phosphorylase-catalyzed enzymatic polymerization of G-1-P from maltooligosaccharide (maltoheptaose, G_7_) was performed in the presence of PTHF dispersed in aqueous buffer, the product was gradually precipitated from the reaction media. The subsequent characterization of the isolated product supported the proposed structure of an amylose-PTHF inclusion complex. Mixing amylose and PTHF in aqueous buffer did not result in the formation of an inclusion complex, strongly suggesting the inclusion occurs during or a result of enzymatic polymerization.

To investigate the effect of the structures of polyethers on the formation of inclusion complexes in vine-twining polymerization, the phosphorylase-catalyzed enzymatic polymerization of G-1-P was conducted using polyethers with different alkyl chain lengths including PTHF (4 methylenes), polyoxetane (POXT, 3 methylenes), and poly(ethylene glycol) (PEG, 2 methylenes) [30]. Consequently, the hydrophobic POXT formed an inclusion complex with amylose, whereas vine-twining polymerization with PEG did not induce inclusion complexation. This was likely due to the hydrophilic nature of PEG, resulting in much less hydrophobic interaction with the amylose cavity. These results highlight the importance of the hydrophobicity of guest polymers in forming inclusion complexes with amylose via vine-twining polymerization.

Hydrophobic polyesters, including poly(ε-caprolactone) (PCL), poly(δ-valerolactone) (PVL), and poly(glycolic acid-*co*-ε-caprolactone) (P(GA-*co*-CL)), have also been used as guest polymers in vine-twining polymerization to form inclusion complexes with amylose, as they contain relatively polar ester bonds in the main-chain [31,32,33]. However, when the homopolyester poly(glycolic acid), was used as a guest polymer it was not able to form an inclusion complex with amylose due to its high crystallinity and low dispersibility in aqueous media.

An inclusion complex was formed via vine-twining polymerization with a hydrophobic poly(ester-ether) (PEE, –CH_2_CH_2_C(C=O)OCH_2_CH_2_CH_2_CH_2_O–) composed of alternating ester and ether linkages [32]. A hydrophobic polycarbonate, poly(tetramethylene carbonate) (PTMC), with relatively polar carbonate bonds, also formed an inclusion complex with amylose via vine-twining polymerization [34]. On the other hand, a hydrophilic poly(ester-ether) (–CH_2_CH_2_C(=O)OCH_2_CH_2_O–) with a short methylene length, could not form an inclusion complex with amylose under the same conditions.

In addition to their inability to form inclusion complexes with hydrophilic polymers, it is difficult to produce inclusion complexes from polymers with strong hydrophobicity owing to aggregation in aqueous buffer. For example, the strongly hydrophobic polyoxepane (6 methylenes), did not form an inclusion complex with amylose via vine-twining polymerization.

**Figure 4:**
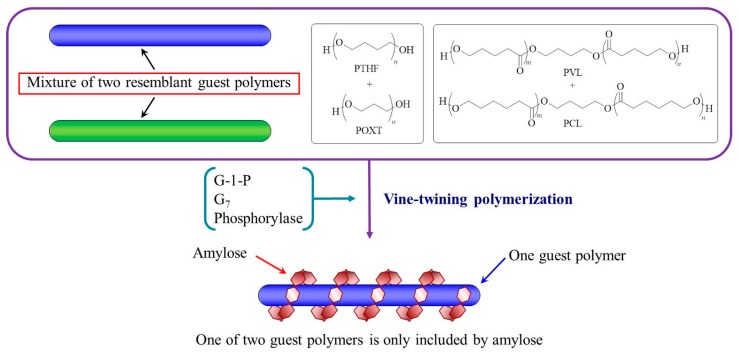
Amylose selectively includes one of two resembling polyethers or polyesters mixture; G-1-P = α-d-glucose 1-phosphate, G_7_ = maltoheptaose, PTHF = polytetrahydrofuran, POXT = polyoxetane, PVL = poly(δ-valerolactone), PCL = poly(ε-caprolactone).

Based on the aforementioned results regarding the formation of inclusion complexes through vine-twining polymerization, we have speculated that moderate hydrophobicity of the guest polymers is required for complexation with amylose. Indeed, amylose exhibits different complexation behaviors depending on subtle changes in the structures of the hydrophobic guest polymers. For example, amylose selectively included PTHF in a mixture of PTHF and POXT in vine-twining polymerization system, owing to the slight difference the hydrophobicity of the potential guest polymers (Figure 4) [35]. Also, in a mixture of PCL and PVL, amylose selectively formed an inclusion complex with PVL during vine-twining polymerization (Figure 4) [36].

Amylose selectively included a specific range of molecular weights of guest polymers in vine-twining polymerization. Synthetic polymers are generally mixtures of different molecular weight analogs, which possess different properties. For example, the molecular weight of PTHF polymers affect its hydrophobicity and water-solubility, where low molecular weight PTHF exhibits good water-solubility, whereas those with larger molecular weight are hydrophobic and insoluble in water. When several vine-twining polymerization systems were studied using PTHFs with different average molecular weights, the specific range of molecular weights of all PTHFs were suitably recognized by amylose to form inclusion complexes [24].

**Figure 5:**
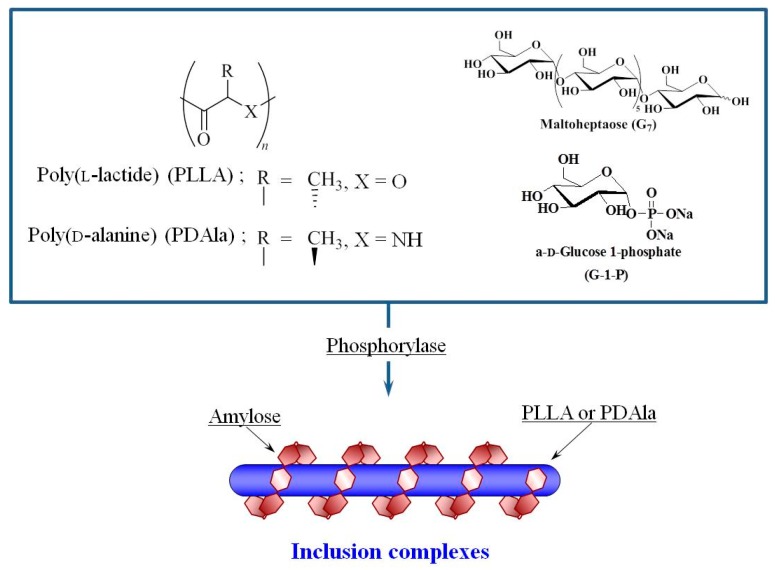
Stereoselective inclusion complexation by amylose in vine-twining polymerization using poly(l-lactide) (PLLA) and poly(d-alanine) (PDAla).

Besides the chemical structure and molecular weight, amylose also showed selectivity towards chirality in guest polymers in vine-twining polymerization. The selective inclusion of chiral molecules by amylose was achieved using chiral polyesters, poly(lactide)s (PLAs) as guest polymers with three stereoisomers, i.e., poly(l-lactide) (PLLA), poly(d-lactide) (PDLA), and racemic poly(dl-lactide) (PDLLA, Figure 5) [37]. When vine-twining polymerization was conducted using PLLA, an inclusion complex was formed, whereas the PDLA and PDLLA polymers did not achieve inclusion complexation.

The selective inclusion based on chirality was also observed in vine-twining polymerization using chiral polyalanine (PAlas) stereoisomers as guest polymers (Figure 5) [38]. An inclusion complex was formed with poly(d-alanine) (PDAla), whereas inclusion complexes were not obtained with poly(l-alanine) (PLAla) or poly(dl-alanine) (PDLAla).

The stereoselective inclusion behavior of amylose toward PLLA and PDAla in vine-twining polymerization can be explained by the helical direction of the host and guest polymers. The left-handed helical conformation of the guest polymers PLLA and PDAla is the same direction as that of the host amylose, resulting in their efficient inclusion. In contrast, the opposite and irregular helical conformations of PDLA/PLAla and PDLLA/PDLAla, respectively, are not suitable for binding by the amylose helix.

## 3. Hierarchical Structured Materials from Amylose-Polymer Inclusion Complexes by Vine-Twining Polymerization

**Figure 6:**
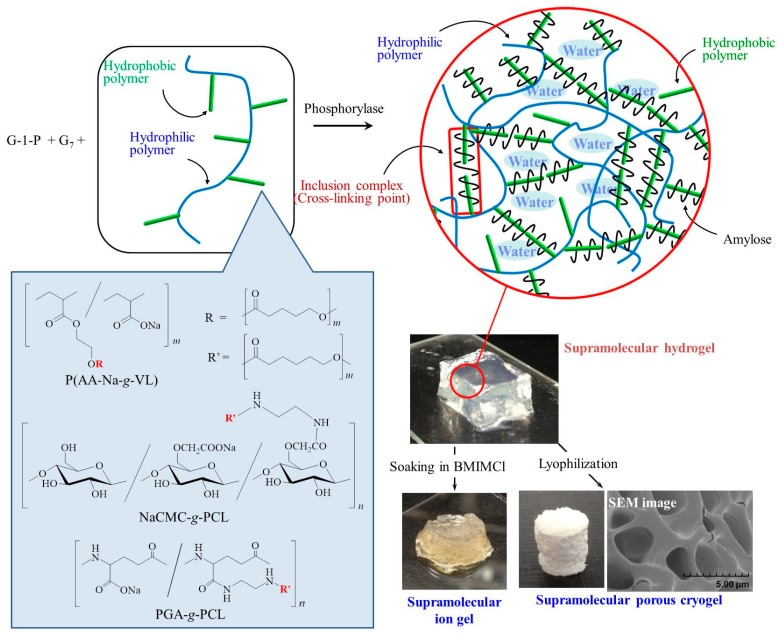
Preparation of hierarchical structured materials by vine-twining polymerization using graft copolymers having hydrophilic main chains and hydrophobic guest graft chains and conversion of supramolecular hydrogel into cryo- and ion gels; G-1-P = α-d-glucose 1-phosphate, G_7_ = maltoheptaose, P(AA-Na-*g*-VL) = poly(acrylic acid sodium salt-*graft*-δ-valerolactone), NaCMC-*g*-PCL = carboxymethyl cellulose sodium salt-*graft*-poly(ε-caprolactone), PGA-*g*-PCL = poly(γ-glutamic acid)-*graft*-poly(ε-caprolactone).

The vine-twining polymerization approach has been applied to the fabrication of hierarchical structured materials, such as gels and films, based on amylose-polymer inclusion complexes [28]. To construct such materials, supramolecular networks, which are hierarchically composed of inclusion complexes as crosslinking points, were designed as vine-twining polymerization products by using graft copolymers with hydrophobic graft chains. Significantly, the enzymatically produced amylose chains include the hydrophobic graft chains as guest polymers to produce inclusion complexes, which act as crosslinking points to hierarchically construct a supramolecular network structure in aqueous media, forming hydrogels (Figure 6). The hydrophobicity of the graft chains as guest polymers is necessary, while the graft copolymer should generally be water-soluble to successfully form hydrogels.

The hierarchical formation of a hydrogel was achieved by the phosphorylase-catalyzed polymerization of G-1-P from G_7_ in the presence of a water-soluble copolymer composed of hydrophobic PVL graft chains, poly(acrylic acid sodium salt-*graft*-δ-valerolactone) (P(AA-Na-*g*-VL)), by vine-twining polymerization (Figure 6) [39]. The enzymatic reaction mixture was completely converted into the hydrogel form. The enzymatically produced amylose included the PVL graft chains to form inclusion complexes as the polymerization progressed, which acted as cross-linking points for hydrogelation. Furthermore, the hydrogels were enzymatically disrupted and reproduced through combination of the β-amylase-catalyzed hydrolysis of amylose and the reformation of amylose by the phosphorylase-catalyzed polymerization.

A film was constructed through the hierarchical formation of a hydrogel by vine-twining polymerization using another graft copolymer, carboxymethyl cellulose sodium salt-*graft*-poly(ε-caprolactone) (NaCMC-*g*-PCL) (Figure 6). The reaction mixture was completely converted into the hydrogel by the vine-twining polymerization [40]. The film was formed by moisturizing the powdered sample prepared by lyophilization of the hydrogel.

The mechanical properties of the hydrogels obtained by vine-twining polymerization using PAA-Na-*g*-PVL and NaCMC-*g*-PCL were insufficient for further applications. To improve the mechanical properties of the hydrogels, poly(γ-glutamic acid) (PGA) was used as the main-chain of a graft copolymer (Figure 6) [41], because its shows better water retention and moisturizing properties. Indeed, vine-twining polymerization using poly(γ-glutamic acid)-*graft*-poly(ε-caprolactone) (PGA-*g*-PCL) resulted in a hydrogel with self-standing properties, indicating much better mechanical properties compared to the aforementioned hydrogels. The prepared hydrogel exhibited macroscopic interfacial healing behavior upon the phosphorylase-catalyzed enzymatic polymerization. The hydrogel formed initially from the vine-twining polymerization was cut into two pieces, and G-1-P and phosphorylase-containing sodium acetate buffer was dropped on the surface of the hydrogels. After the surfaces were placed in contact with one another, the materials were left standing for enzymatic polymerization. Consequently, the two hydrogel pieces were fused at the contacted area. Such behavior of the hydrogels on a macroscopic level was induced by the complexation of the enzymatically produced amyloses with the PCL graft chains at the interface. In addition, a porous cryogel and an ion gel were obtained by lyophilization and soaking of the hydrogel in an ionic liquid of 1-butyl-3-methylimidazolium chloride (BMIMCl) (Figure 6).

**Figure 7:**
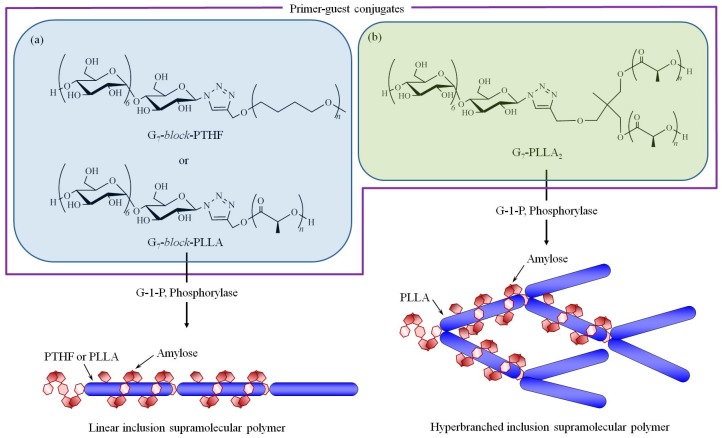
Formation of (**a**) linear and (**b**) hyperbranched supramolecular polymers by vine-twining polymerization using primer–guest conjugates; G-1-P = α-d-glucose 1-phosphate, G_7_-*block*-PTHF = maltoheptaose-*block*-polytetrahydrofuran, G_7_-*block*-PLLA = maltoheptaose-*block*-poly(l-lactide), G_7_-PLLA_2_ = branched maltoheptaose-(poly(l-lactide))_2_.

Supramolecular polymers composed of amylose-PTHF and amylose-PLLA inclusion complexes were dynamically formed by vine-twining polymerization using primer–guest conjugates, i.e., maltoheptaose-*block*-polytetrahydrofuran (G_7_-*block*-PTHF) and maltoheptaose-*block*-poly(l-lactide) (G_7_-*block*-PLLA, Figure 7a) [42,43]. In these systems, an enzymatically propagating amylose chain included a PTHF or PLLA segment of another conjugate, whereby consecutive inclusion led to the formation of linear supramolecular polymers.

Vine-twining polymerization using a branched maltoheptaose-(poly(l-lactide))_2_ (G_7_-PLLA_2_) conjugate resulted in a hyperbranched supramolecular polymer (Figure 7b) [44]. The hyperbranched product formed an ion gel with BMIMCl, which was further converted into a hydrogel upon exchange of the dispersion media by soaking in water. Lyophilization of the resulting hydrogel produced a porous cryogel.

The relative chain orientation of amylose and PLLA in the supramolecular polymers was investigated using two G_7_-*block*-PLLA conjugates, which were composed of a G_7_ moiety interconnected to the carboxylate or hydroxy terminus of PLLA [45]. Enzymatic polymerization in the presence of the two PLLA conjugates formed amylose-PLLA supramolecular polymers by vine-twining polymerization. This suggested that, regardless of the chain orientation of PLLA, the amylose cavity recognized the PLLA segment for complexation. Conversely, the phosphorylase-catalyzed enzymatic polymerization in the presence of the two G_7_-*block*-PDLA conjugates under similar conditions only resulted in the formation of amylose-PDLA diblock copolymers, which did not form an inclusion complex structure. These results indicated that chirality in PLAs affected the inclusion behavior of the amylose cavity, irrespective of the PLA chain orientation. The left-handed helices of both the amylose and PLLA induce inclusion complexation, whereas complexation was not significantly affected by the orientation of the methyl substituents in PLA, which oppositely change according to the relative chain orientation.

## 4. Conclusions

In this review, we presented our studies on the precision preparation of amylose-polymer inclusion complexes through the phosphorylase-catalyzed enzymatic polymerization of G-1-P in the presence of synthetic hydrophobic polymers by vine-twining polymerization. The results of the vine-twining polymerization study suggested that amylose exhibited different inclusion behaviors depending on the specific interactions with the guest polymers according to subtle changes in their structures. Moreover, hierarchical structured materials could be dynamically fabricated by vine-twining polymerization using designed graft copolymers composed of hydrophilic main chains and hydrophobic guest graft chains. Vine-twining polymerization using guest-primer conjugates afforded the dynamic formation of the supramolecular polymers composed of a continuum of inclusion complexes. Because of the production of a structurally defined amylose host by phosphorylase catalysis, the precision preparation of controlled amylosic host-guest polymeric complexes was achieved. Therefore, the vine-twining polymerization method can be applied to the production of additional amylosic inclusion complexes with regularly controlled nanostructure, and will contribute to further developments of host-guest chemistry in the future.
